# Diversity and potential activity of methanotrophs in high methane-emitting permafrost thaw ponds

**DOI:** 10.1371/journal.pone.0188223

**Published:** 2017-11-28

**Authors:** Sophie Crevecoeur, Warwick F. Vincent, Jérôme Comte, Alex Matveev, Connie Lovejoy

**Affiliations:** 1 Département de Biologie, Centre d’études nordiques (CEN) and Takuvik Joint International Laboratory, Université Laval, Québec, Québec, Canada; 2 Institut de Biologie Intégrative et des Systèmes, Université Laval, Québec, Québec, Canada; 3 Québec-Océan, Université Laval, Québec, Québec, Canada; Purdue University, UNITED STATES

## Abstract

Lakes and ponds derived from thawing permafrost are strong emitters of carbon dioxide and methane to the atmosphere, but little is known about the methane oxidation processes in these waters. Here we investigated the distribution and potential activity of aerobic methanotrophic bacteria in thaw ponds in two types of eroding permafrost landscapes in subarctic Québec: peatlands and mineral soils. We hypothesized that methanotrophic community composition and potential activity differ regionally as a function of the landscape type and permafrost degradation stage, and locally as a function of depth-dependent oxygen conditions. Our analysis of *pmoA* transcripts by Illumina amplicon sequencing and quantitative PCR showed that the communities were composed of diverse and potentially active lineages. Type I methanotrophs, particularly *Methylobacter*, dominated all communities, however there was a clear taxonomic separation between the two landscape types, consistent with environmental control of community structure. In contrast, methanotrophic potential activity, measured by *pmoA* transcript concentrations, did not vary with landscape type, but correlated with conductivity, phosphorus and total suspended solids. Methanotrophic potential activity was also detected in low-oxygen bottom waters, where it was inversely correlated with methane concentrations, suggesting methane depletion by methanotrophs. Methanotrophs were present and potentially active throughout the water column regardless of oxygen concentration, and may therefore be resilient to future mixing and oxygenation regimes in the warming subarctic.

## Introduction

Methane emissions to the atmosphere are the net result of microbial methanogenesis and methanotrophy, and in many environments, the activity of aerobic methanotrophic bacteria (methanotrophs) may impose a strong control on methane emission flux rates [1]. Methanotrophs have the distinctive ability to use methane as their sole source of carbon and energy [2] and have the capacity to oxidize up to 60% of the yearly methane produced at a global scale [3]. In rice fields, the proportion of methane oxidized by methanotrophs varies from 20% to 90% [4–6]. In wetlands and freshwater ecosystems, up to 95% of the methane produced is consumed by oxidation [7–9], and in a boreal lake, up to 88% of the methane diffusing into the water column was estimated to be consumed by methanotrophs [10,11]. Less is known about the importance of methanotrophy in Arctic and subarctic ecosystems. In High Arctic soils, methanotrophs in the upper profile appear to be sufficiently active to fully consume all the methane produced, and the soils are net methane sinks [12], while northern lakes appear in general to be net sources of methane [13,14].

Methanotrophs require the presence of oxygen as an electron acceptor for methane oxidation [15], and in lakes, methanotrophs would be logically more abundant in the oxic/anoxic interface where both methane and oxygen are available [2]. However, they can also be found homogeneously distributed throughout the water column [16], and sometimes in unexpectedly higher concentrations in anoxic bottom waters [17]. Given these disparate observations, it is still unclear to what extent methanotrophs are able to oxidize methane under different in situ oxygen conditions.

The application of functional genomics to ecosystems provides a means to establish links between microbial diversity and biogeochemical processes [18], including for microbes involved in the methane cycle. The functional gene coding for the α-subunit of particulate methane mono-oxygenase (*pmoA*), is a phylogenetically useful marker for enzymes involved in bacterial methane oxidation, and can be used to assess methanotroph community composition and activity [19–21]. Methanotrophs are commonly separated into two types based on phylogenetic and metabolic differences [22]. Type I methanotrophs (gamma-proteobacteria) use the ribulose monophosphate (RuMP) pathway for carbon assimilation while Type II methanotrophs (alpha-proteobacteria) use the serine pathway. Type I taxa are sometimes further divided into Type Ia (*Methylobacter*-related) and Type Ib (*Methylococcus*-related) based on their phylogenetic affiliation [23]. Recently, new groups of methanotrophs have been discovered that are phylogenetically distant from Type I and II: the genus *Crenothrix*, which belongs to the gamma-proteobacteria but has a phylogenetically divergent *pmoA* [24]; the verrucomicrobial order Methylacidiphilales [25]; and the NC10 phylum that has the ability to couple methane oxidation with denitrification [26].

Lakes and ponds, which are a characteristic feature across northern landscapes, are strong emitters of methane to the atmosphere [14,27]. Emission rates are especially high from thermokarst lakes and ponds (thaw ponds) in subarctic permafrost peatlands [28], but little attention has been given to their methane oxidation potential. Thaw ponds in general are especially sensitive to climate warming. In some areas, they are draining and infilling, while in other northern regions they are expanding (see [29] and references therein). Overall, thawing and collapse of permafrost (thermokarst erosion), has an impact on the size and abundance of such ponds, and large quantities of organic carbon that were previously stored in the frozen soils are being mobilized across these landscapes [30].

In the Quebec subarctic region, permafrost thaw ponds have different geomorphological origins that may influence methanotrophic community composition and activity. First, the quality and the quantity of the carbon pool in the ponds change as a function of permafrost degradation and the landscapes surrounding the ponds, with two distinct landscape types: peatlands with raised mounds of organic material (palsas) and shrub-tundra landscapes with raised mounds of inorganic soils (lithalsas). These different landscapes affect the community composition of methanogenic Archaea [31] and also methane oxidation rates [32], but the influence on methanotrophic communities has been little explored. These landscapes lie in the transition zone at the southern limit of the current permafrost range, and the ice-rich mounds are thawing and degrading rapidly. This thermokarst activity is resulting in the production and expansion both of palsa and lithalsa thaw ponds, which differ in their limnological properties such as dissolved organic carbon concentration and pH [33–35]. An unusual feature of both types of subarctic ponds is their tendency to be highly stratified during summer, with marked gradients of temperature and oxygen down through the water column [36]. This stratification is likely to have an impact on methane production (methanogenesis) and loss processes (methanotrophy) that are both dependent on temperature and oxygen availability. Furthermore, the high concentrations of suspended particulates in these water favours particle-attached bacterial populations over free-living cells [37], but the taxonomic composition of the two communities has not been established.

Given the heterogeneity of conditions in subarctic thaw ponds at local and regional scales, our goal in the present study was to identify the factors that influence the composition and potential activity of their methanotrophic communities. We conducted this research by way of phylogenetic and quantitative PCR (qPCR) analysis of *pmoA* transcripts in ponds across a gradient of permafrost degradation gradient, and in the two ecosystem types: organic-rich palsa ponds and mineral-rich lithalsa ponds. We hypothesized that the methanotrophic community and their potential activity differ as a function of landscape type, extent of permafrost degradation and depth in the ponds. We surmised that methanotrophic activity down the water column would be determined by oxygen availability, limiting *pmoA* transcription to the oxygenated surface waters. We additionally tested the difference in *pmoA* phylogenies between free-living and attached methanotrophic communities, given the importance of particles for bacterial activity in these waters.

## Materials and methods

### Study site and sampling

**Fig 1:**
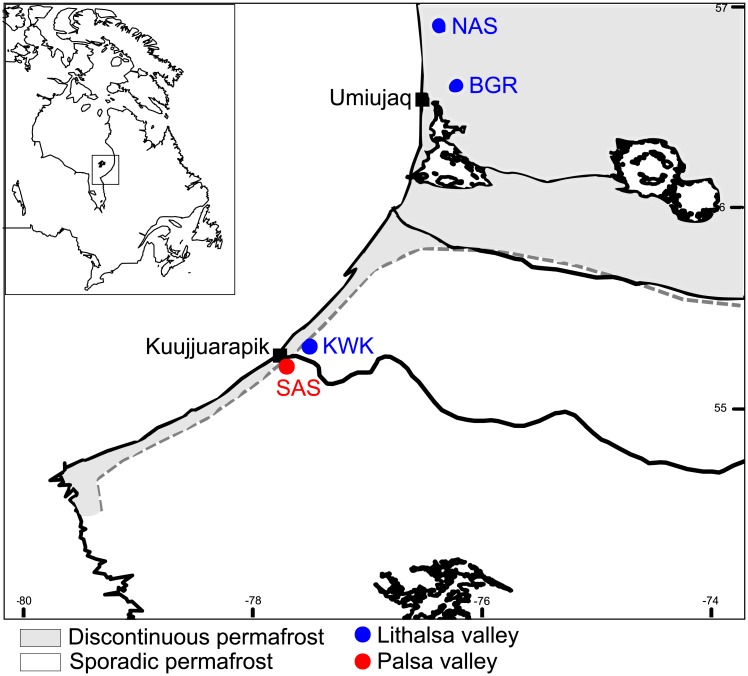
The eastern Hudson Bay region around the villages of Kuujjuarapik and Umiujaq in subarctic Quebec, Canada. The locations of the four sampled valleys are shown with red (palsa) and blue (lithalsa) dots across the permafrost degradation gradient. Map created in R with the open-access databases "worldHires" https://www.evl.uic.edu/pape/data/WDB/ and data from the Digital Chart of the World downloaded from DIVA-GIS (http://www.diva-gis.org).

Water samples were collected during two field expeditions, 1 to 13 August 2012 and 31 July to 19 August 2013, from four subarctic valleys in northern Québec (Fig 1). The Kwakwatanikapistikw River valley (hereafter KWK valley; lat. 55° 19.95′ N, long. 77° 30.13′ W) and Sasapimakwananisikw River valley (SAS valley; lat. 55° 13′ N; long 77° 42′ W) are located in the sporadic permafrost region, where permafrost underlies less than 50% of the terrain, and the nearest village is Whapmagoostui-Kuujjuarapik. The Sheldrake River valley (BGR; lat. 56° 37′ N; long. 76° 13′ W) and Nastapoka River valley (NAS; lat. 56° 55′ N, long. 76° 22′ W) are situated 100 km north of the two other valleys, in the discontinuous permafrost region, close to the village of Umiujaq, Quebec (details in Crevecoeur et al. [34]). The SAS valley ponds are the result of thawing of palsas, peat-rich permafrost mounds [38], while NAS, BGR and KWK ponds originated from thawing of lithalsas, mineral permafrost mounds [39–41]. All required permits and permission to work in the two regions were obtained from the local village and regional Nunavik offices. Surface (0.2 m below surface) and bottom (0.2 m above sediments) water samples were taken from 12 ponds across the four valleys, which resulted in a total of 24 samples (Table 1).

**Table 1:** Details of the ponds sampled during the 2012 and 2013 field campaigns and the samples used for physico-chemical (P-C) and molecular (Illumina and qPCR) analyses.

Nomenclature	Valley	Pond	Depth	Year	Datasets
NASH-S	NAS	H	Surface	2012	P-C, qPCR*
NASH-B	NAS	H	Bottom	2012	P-C, Illumina, qPCR
BGR1-S	BGR	1	Surface	2013	P-C, Illumina, qPCR
BGR1-B	BGR	1	Bottom	2013	P-C, Illumina, qPCR
BGR2-S	BGR	2	Surface	2012	P-C, Illumina, qPCR
BGR2-B	BGR	2	Bottom	2012	P-C, Illumina, qPCR
KWK1-S	KWK	1	Surface	2012	P-C
KWK1-B	KWK	1	Bottom	2012	P-C, Illumina, qPCR
KWK6-S	KWK	6	Surface	2012	P-C
KWK6-B	KWK	6	Bottom	2012	P-C, Illumina, qPCR
KWK12-S	KWK	12	Surface	2013	P-C, qPCR*
KWK12-B	KWK	12	Bottom	2013	P-C, qPCR*
KWK23-S	KWK	23	Surface	2012	P-C
KWK23-B	KWK	23	Bottom	2012	P-C, Illumina, qPCR
SAS1A-S	SAS	1A	Surface	2013	P-C, Illumina*,qPCR
SAS1A-B	SAS	1A	Bottom	2013	P-C, Illumina*, qPCR
SAS1B-S	SAS	1B	Surface	2013	P-C
SAS1B-B	SAS	1B	Bottom	2012	P-C, Illumina, qPCR
SAS2A-S	SAS	2A	Surface	2012	P-C, Illumina, qPCR
SAS2A-B	SAS	2A	Bottom	2012	P-C, Illumina, qPCR
SAS2A-Sb	SAS	2A	Surface	2013	P-C, qPCR
SAS2A-Bb	SAS	2A	Bottom	2013	P-C, qPCR
SAS2B-S	SAS	2B	Surface	2013	P-C, Illumina, qPCR
SAS2B-B	SAS	2B	Bottom	2013	P-C, Illumina, qPCR

*Only for the small fraction

Profiles of temperature, dissolved oxygen (DO), and pH were taken with a YSI 600R multiparameter probe (Yellow Springs Instruments); the detection limit for dissolved oxygen with this probe is 0.2 mg L^-1^. Bottom water samples were collected using a horizontally mounted Van Dorn bottle (Wilco) and immediately transferred to acid washed 4-L Cubitainers^™^ that were rinsed with sample water prior to filling. The Cubitainers^™^ were overfilled to avoid oxygenation, capped, placed in coolers and brought back to the laboratory. Sub-samples for methane concentrations were measured with the headspace technique as described in Matveev et al. [28], while samples for physico-chemical analysis (DOC, TSS and TP) were processed as in Crevecoeur et al. [34].

### RNA sample preparation and sequencing

The water for RNA analysis was prefiltered through a 20 μm mesh (Nitex) and then sequentially filtered through a 3 μm Nuclepore^™^ polycarbonate (PC) filter and a 0.2 μm Sterivex^™^ unit (Millipore) to separate large (particle-attached; >3 μm) and small (free-living; 0.2–3 μm) fractions of planktonic micro-organisms. After one hour of filtration, the filtration was stopped and filters were conserved in RNAlater (Life Technologies) and subsequently stored at -80°C until extraction. RNA was extracted with the AllPrep DNA/RNAMini Kit (Qiagen) modified to include an additional step using polyvinylpyrrolidone (PVP, Alfa Aesar) to minimize potential PCR inhibition [42]. RNA was converted to cDNA using the High Capacity cDNA Reverse Transcription Kit (Applied Biosystems-Ambion). The quantity and quality of cDNA was checked on a 1% agarose gel; samples with sufficient cDNA for further processing (Table A in S1 File) were stored at -80°C.

Amplification of *pmoA* was performed with a two-step dual-indexed PCR approach modified for Illumina^®^ sequencing, with two consecutive sets of primers (Table B in S1 File). In the first step, the gene specific portion was fused to the Illumina^®^ TrueSeq sequencing primers and PCR was carried out in a total volume of 25 μL that contained HF buffer 1X (NEB), 0.25 μM of each primer, 200 μM of each dNTPs (Life Technology), dimethylsulfoxide (DMSO, NEB) at a final concentration of 3%, 1 U of Phusion^®^ High-Fidelity DNA polymerase (NEB) and 1 μL of template cDNA. To minimise primer bias, two more reactions with 5 and 10 fold diluted template were also carried out for each sample. Temperature and duration of thermal cycling were started with an initial denaturation at 98°C for 30 s followed by 35 cycles of denaturation at 98°C for 10 s, annealing at 56°C for 30 s, extension at 72°C for 30 s and a final extension at 72°C for 300 s. The three dilution reactions were pooled together and purified using the Axygen PCR cleanup kit. The quality and quantity of the purified PCR product were checked on a 1% agarose gel. Then 50 to 100-fold dilutions of this purified product was used as a template for a second PCR step to add barcodes (dual-indexed). The *pmoA* marker was not successfully amplified from some cDNA samples and these were excluded from further processing and sequence analysis (Table 1). This second PCR was done in triplicate under the same conditions as the first PCR but with 15 cycles. Triplicates were pooled and purified as above, and then quantified spectrophotometrically with a NanoDrop^™^ 1000 (Thermo Fisher Scientific). Barcoded amplicons were pooled in equimolar concentrations for pair-end sequencing on the Ilumina MiSeq at the Plateforme d’Analyses Génomiques (IBIS, Université Laval, Québec, Canada). The raw Illumina reads have been deposited in the NCBI sequence read archive (SRA) under accession number SRP091008.

### Sample processing for qPCR

Standards were prepared from PCR product produced under the same conditions as the first reaction PCR of the dual-indexed PCR for Illumina sequencing. Triplicate samples of PCR product were purified with a Feldan (Bio-Basics) PCR purification kit. Amplicons were cloned using a Stratagene cloning kit following the manufacturer’s instructions, with the following modifications: a polyA tail was added to the amplicons using Feldan polymerase and buffer and dATPs (final concentration 0.175 nM) and 26 to 30 ng were used for the ligation reaction. Transformed cells were incubated 1 h in SOC medium at 37°C for recovery before plating on agar plates. Positive clones were incubated again in LB media containing 7% glycerol for 16h at 37°C. To verify that the target gene was amplified, the T3/T7 PCR reaction was performed on the clones and 11 of them were sent for Sanger sequencing at the Sequencing Center of Laval University Hospital Center (CHUL; Quebec City, QC, Canada). Standards were then prepared by amplifying a positive clone with T3 and T7 primers. Amplicons were checked on a 1% agarose gel and the band corresponding to the size of the target amplicon (around 700bp) was cut and purified with a gel purification kit (Qiagen). Another T3/T7 PCR was performed on the purified amplicon and was purified with a PCR purification kit (Feldan). Concentrations were measured with the NanoDrop^™^ spectrometer. Each reaction (standard and samples) was run in triplicate. Standards for calibration were diluted 10 times to ensure measurements from 10^7^ to 10^1^ copies μL^-1^. Potential inhibition was checked by running 10 and 100 fold dilutions of the sample, which covered the expected copy number range. qPCR reactions for standards and samples were performed in 20 μL reactions containing 5 μL of template, 500 μM of each primer (PmoA169F and PmoA661r), 1X Ssofast EvaGreen^™^ Supermix and nuclease free water on a Chromo4 thermal cycler (Bio-Rad) with the following steps: initial denaturation (30 s at 95°C) and then 40 cycles of denaturation (5 s at 95°C), annealing (30 s at 55°C) and elongation (20 s at 72°C). At this step no transcripts were detected in some samples (Table 1).

### Sequence processing and analysis

*PmoA* reads from the Illumina amplicon sequencing were first assembled using the usearch command “fastq_mergepairs” from UPARSE [43] and then further analyzed using the FunGene pipeline [44]. Reads shorter than 400 bp were discarded and chimeras were checked and removed with UCHIME [43]. Sequences were translated to amino acids and compared to the *pmoA* reference sequence with FrameBot for detecting frameshift errors and sequences with inframe STOP codons, which were removed. Remaining high quality sequences were aligned with HMMER3 and then clustered at 93% similarity, which has been identified as the threshold that corresponds to 97% sequence similarity for 16S rRNA [20,45]. A custom *pmoA* database was constructed by downloading *pmoA* sequences from the Functional Gene Repository v.8.0. Reference sequences were checked against the NCBI nr database and the reference database was manually inspected to ensure all types of known methanotrophs were represented. Taxonomic affiliation of the representative sequences of *pmoA* operational taxonomic units (OTUs) was determined in QIIME using the assign_taxonomy.py command [46]. For unassigned sequences, a Neighbour Joining 1000 bootstraps tree following the Poisson model was constructed to allow assignation of sequences to Type Ia, Type Ib or Type II methanotrophs by phylogenetic affiliation. Rarefaction curves of OTUs were based on 93% similarity using the command alpha_rarefaction.py available in QIIME. The dataset was re-sampled 100 times to standardize to 37,000 reads per sample, which corresponded to the minimum number of reads in a sample minus 10%, using the command multiple_rarefaction_even_depth.py in QIIME. The community data matrix was square root transformed before running the Bray-Curtis dissimilarity measure. The Ward method was used for clustering and Bray-Curtis distances were squared as recommended in Murtagh and Legendre [47]. A principal component analysis (PCA) was carried out on the environmental variables with the function rda in the Vegan package [48] and missing values were imputed using the function impute PCA in the missMDA package [49]. Variables that were not normally distributed were log transformed to meet the test assumptions. Statistical differences between valleys and depth in terms of the physico-chemical properties of the ponds were tested with a multivariate analysis of variance (MANOVA).

Testing for differences in the qPCR data required a non-parametric test since the values did not meet normality assumptions even after log transformation. The Kruskal-Wallis test was then used to assess differences in methanotrophic potential activity between valleys, depth and size fraction. To compare with the β-diversity pattern, 15 samples (of the 24 total) that had corresponding Illumina reads were used in this analysis (Table 1). Surface samples that fell below the limit of detection for the qPCR measurements were considered as missing data.

To evaluate which environmental variables and groups of methanotrophs (X-variables) contributed to the potential activity of methanotrophs (Y-variable), we used partial least squares regression (PLS) in the R package mixOmics [50], which transforms X into latent variables to explain the maximum variance in Y. For this analysis, OTUs were binned into phylogenetic groups (genera), and the unclassified Methylococcaceae were phylogenetically assigned to Type Ia and Type Ib following their placement in the phylogenetic tree. The PLS analysis has the advantage of being robust despite collinearity and missing data [51]. The missing values in X were replaced by values from model prediction using the NIPALS algorithm. Prior to analysis, the Y-variable data and the environmental data that were not normally distributed were log transformed. Methanotroph data that were zero-inflated were fourth-root transformed, as recommended in Wold et al. [51]. The results of the PLS were then examined via correlation plots to identify variables correlated with the expression of the *pmoA* gene. The relationship between the small fraction of the qPCR data and CH_4_ concentration was assessed for the bottom samples. The qPCR data were log transformed to meet normality assumptions. Pearson correlation values were then calculated using the Vegan package in R [48].

## Results

### Physico-chemical properties

**Fig 2:**
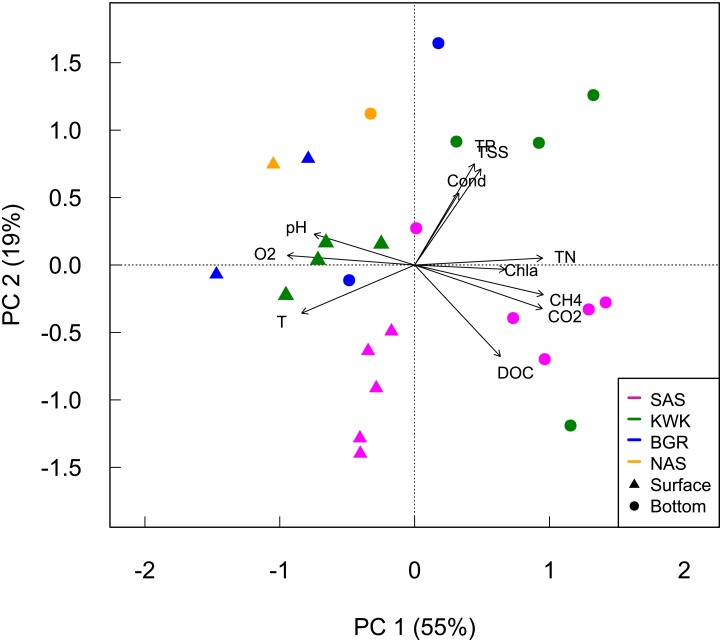
Principal component analysis of the environmental variables. Temperature (T), dissolved oxygen (O2), pH, total phosphorus (TP), total suspended solid (TSS), conductivity (Cond), Chlorophyll *a* (Chla), total nitrogen (TN), methane (CH_4_), carbon dioxide (CO_2_) and dissolved organic carbon (DOC) are represented for the 9 sampled ponds. Colors distinguish the different valleys and the shapes distinguish surface (triangles) and bottom samples (circles).

The surface waters of the ponds were warmer, with higher oxygen concentrations and pH values compared to their bottom waters (Fig 2). Ponds BGR-1 and BGR-2 were oxygenated throughout the water column, while the other ponds were hypoxic (DO < 3 mg L^-1^) to anoxic at the bottom. Ponds from the northern BGR and NAS valleys had higher pH values (from 7 to 8), while pH was more acidic in the southern valleys (from 5 to 6). Bottom waters of the ponds in the KWK and SAS valleys had higher CO_2_, CH_4_ and nutrient concentrations, with highest concentrations of total nitrogen and phosphorus in the bottom of KWK23: 2.7 mg N L^-1^ and 170.5 μg P L^-1^. The TSS values followed the same trend, with the highest value at the bottom of the KWK1 pond of 140.8 mg L^-1^. The difference between lithalsa and palsa ponds was most pronounced in terms of DOC concentration: ponds from the SAS peatland valley contained higher DOC concentrations, which ranged from 9.6 mg L^-1^ at the bottom of SAS1A to 18.9 mg L^-1^ for the bottom of SAS2A (Fig 2, Table C in S1 File). In terms of limnological properties, the ponds differed significantly among valleys and depths (MANOVA, respectively p = 0.03 and p = 0.008).

### Methanotroph community composition

A total of 1,850,808 *pmoA* reads were retained after quality control and cleaning, and corresponded to a total of 985 OTUs at 93% similarity. The semi-parabolic profile of the rarefaction curve suggested a good representation of the diversity of methanotrophs within each valley (Fig A in S1 File). The SAS valley ponds reached plateaus at a higher level than for those in the other valleys. On average, SAS valley samples plateaued above 300 OTUs, KWK and BGR valleys around 200 OTUs, and the NAS valley around 150 OTUs. However, there were no significant differences (p > 0.05) in the Shannon and Chao1 indices of alpha-diversity between the valleys.

**Fig 3:**
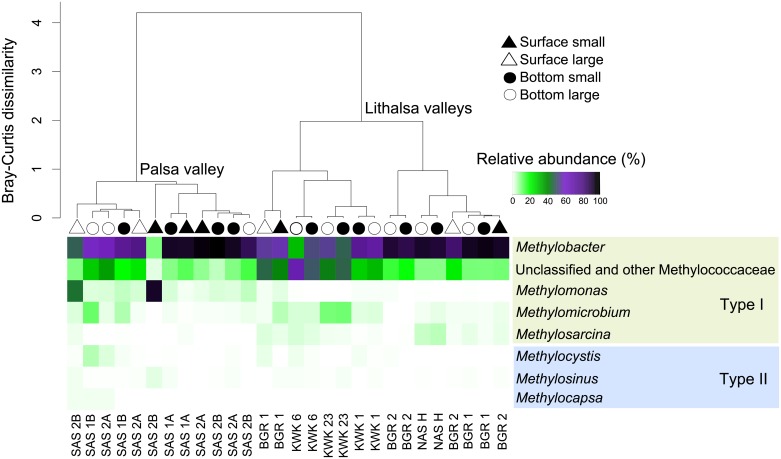
Bray-Curtis dissimilarity cluster analysis of the methanotroph communities. Surface samples are represented by triangles and bottom samples by circles, either filled (small fraction) or open (large fraction). The heatmap shows the methanotroph community composition.

The taxonomy of potentially active methanotrophs also differed markedly between the palsa and lithalsa valleys (Fig 3): the permutation test (9999 permutations) showed that the community structure differed significantly among the different valleys (p = 0.001). There was no significant difference between the two depths of sampling or the two filter size fractions (p > 0.05) for all the valleys considered together. However, within the palsa ponds, there was a significant difference between the small and the large fractions (p = 0.015).

All of the pond communities were dominated by Type I methanotrophs (Fig 3). The most abundant transcripts in the potentially active community belonged to the genus *Methylobacter*, which accounted for up to 92% of the 37,000 subsampled reads in the small fraction from the bottom of SAS2B. The group ‘unclassified’ and other Methyloccocaceae contained sequences that were phylogenetically assigned to Type Ia (> 1% of the reads at all sites) and Type Ib (< 1% of the reads for most sites), and to other genera such as *Methylovulum*, *Methylosoma* and *Methylococcus*, which represented less than 1% of reads in each sample and were then grouped with unclassified and other Methylococcaceae. The genus *Methylomonas* had a low relative abundance in the lithalsa ponds. It was better represented in the palsa ponds, and abundant in the surface of both the small and large fractions in SAS2B (87% and 41% of the reads, respectively). The genus *Methylomicrobium* was present in each sample, ranging from less than 1% in the large fraction of BGR2 bottom to 11% of the reads in the large fraction of SAS1B bottom. Unlike *Methylomonas*, the genus *Methylosarcina* was better represented in the lithalsa sites and reached its highest abundance in the small and large fractions from the bottom of NASH ponds (5% and 4% of the reads, respectively).

Type II methanotroph transcripts were poorly represented in the potentially active community and showed less diversity relative to Type I taxa. Only three Type II genera were identified. *Methylocystis* had highest transcript contribution to the potentially active community (5% of the reads) in the large fraction from the SAS1B bottom, but was absent from several samples in the palsa and lithalsa valleys. *Methylosinus* was present in lower abundance, with a maximum of 2% of the reads in the small fraction from the SAS2B surface waters. Finally, *Methylocapsa* was found in SAS1B, SAS2A and SAS2B, always at less than 1% of the reads.

### Methanotroph potential activity

The potential activity of the methanotroph community was inferred by the concentration of *pmoA* transcripts per mL of sample water (Table 2). Regression coefficients of the calibration curves were between 0.991 and 0.999, qPCR efficiency was between 82% and 115% and the limit of detection was 10 *pmoA* transcript copies. The number of *pmoA* transcripts per mL varied from 2.2 × 10^2^ for the surface small fraction from BGR1 to 7.6 × 10^6^ for the bottom large fraction from SAS1B. There were no significant differences in the number of *pmoA* transcripts among valleys or fractions (Kruskal-Wallis test, p >0.05), but measurements were higher in 2012 compared to 2013. The results showed potential methanotrophic activity not only in the oxygenated surface waters but also in the hypoxic to anoxic bottom waters of the ponds. This included the bottom waters SAS2B and SAS2Ab, where oxygen concentrations were below our limit of detection (< 0.2 mg L^-1^; Table 2, Table C in S1 File).

**Table 2:** Concentrations of *pmoA* transcripts, methane and oxygen and methane in the sampled thaw ponds.

Pond	*pmoA* transcripts mL^-1^		CH_4_ (μM)	O_2_ (mg L^-1^)
	Small fraction (<3μm)	Large fraction (>3μm)		
BGR1-S	2.2 × 10^2^	4.3 × 10^3^	1.1	9.9
BGR1-B	5.2 × 10^3^	3.4 × 10^3^	-	3
BGR2-S	4.4 × 10^6^	3.8 × 10^5^	0.4	9.4
BGR2-B	1.6 × 10^6^	9.4 × 10^4^	2.6	3.5
NASH-S*	1.9 × 10^5^	-	0.1	9.7
NASH-B	1.6 × 10^3^	1.6 × 10^4^	-	1.7
KWK1-B	1.0 × 10^6^	2.1 × 10^5^	-	0.5
KWK6-B	6.4 × 10^5^	3.6 × 10^5^	-	1.8
KWK12-S*	1.7 × 10^2^	-	0.2	8.5
KWK12-B*	6.2 × 10^2^	-	351.3	0.3
KWK23-B	1.3 × 10^6^	4.3 × 10^5^	-	0.4
SAS1A-S	1.6 × 10^3^	3.9 × 10^2^	-	-
SAS1A-B	4.1 × 10^2^	4.9 × 10^2^	-	-
SAS1B-B	2.6 × 10^5^	7.6 × 10^6^	-	1.7
SAS2A-S	2.8 × 10^4^	2.2 × 10^6^	3.4	5.8
SAS2A-B	1.3 × 10^6^	5.9 × 10^6^	101.7	0.3
SAS2A-Sb*	6.6 × 10^3^	5.0 × 10^3^	1.9	2.2
SAS2A-Bb*	1.5 × 10^3^	2.5 × 10^4^	322.9	0.2
SAS2B-S	3.1 × 10^3^	1.4 × 10^4^	3	5.2
SAS2B-B	2.8 × 10^4^	2.6 × 10^5^	292	0.2

-: no data.

*no Illumina sequences available for these samples

**Fig 4:**
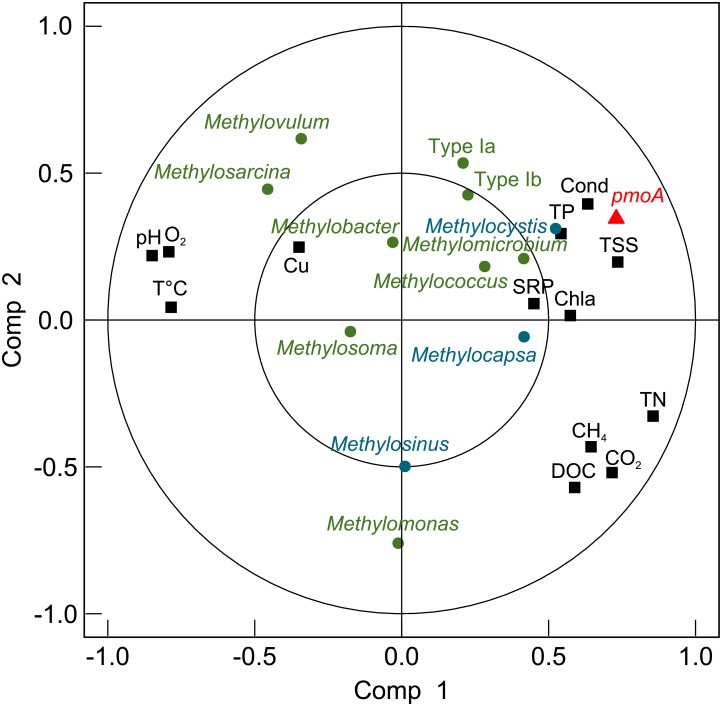
Correlation plot of the PLS analysis to evaluate the potential influence of environmental variables and methanotrophic genera on the potential activity of methanotrophs. The environmental variables were: conductivity (Cond), total phosphorus (TP), total suspended solid (TSS), Chlorophyll *a* (Chla), soluble reactive phosphorus (SRP), total nitrogen (TN), carbon dioxide concentration (CO_2_), methane concentration (CH_4_), dissolved organic carbon (DOC), oxygen concentration (O_2_), temperature (T°C) and pH.

**Fig 5:**
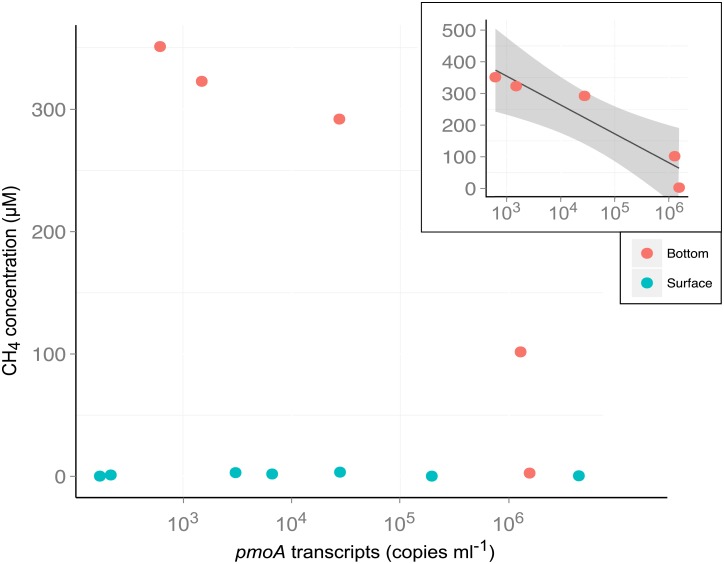
Concentration of *pmoA* transcripts in the small fraction as a function of methane concentration. The inserted graph shows the correlation between methane (μM) and *pmoA* transcript concentrations (copies mL^-1^) in the small fraction of the bottom samples; the shading represents ± 95% confidence intervals.

The factors that influence methanotroph potential activity were evaluated with a PLS analysis. The first two latent variables of the PLS cumulatively explained 61% of the variation (Fig 4). The concentration of *pmoA* was mostly related to conductivity, total phosphorus, total suspended solids and the abundance (number of reads) of *Methylocystis*. The relationship between CH_4_ concentration and potential methanotroph activity varied with cell fraction and depth. The large fraction at both depths and the surface samples for the small fraction showed no clear trend. However the small fraction in the bottom samples showed a strong, negative, nonlinear relationship between *pmoA* transcripts (log transformed) and CH_4_ concentrations (R^2^ = 0.986, p = 0.002; Fig 5).

## Discussion

### Physico-chemical properties

The subarctic permafrost thaw ponds sampled here varied greatly in their limnological properties, with large differences even among ponds located in the same valley. All of the ponds displayed strong physico-chemical gradients down their shallow water columns during this period of summer stratification. These waterbodies annually go through two mixing periods, first in early spring (May) and again during the fall (September). However, such mixing does not always extend to the bottom [52,53]. KWK valley bottom water was reported to be hypoxic to anoxic since 2006 [52,54]. The strong summer stratification and physico-chemical gradients in the water column with hypoxic to anoxic bottom waters imply that the ponds in the KWK and SAS valleys would be favourable environments for both methane synthesis and oxidation, with methanogenesis occurring in the bottom of the pond, and methane consumption higher in the water column where more oxygen is available. These northern ponds also experience large interannual variations; the bottom waters of BGR1 were anoxic in 2005 [36] and 2011, but not in 2012 and 2013. The limnological properties of the ponds were consistent with their origins as either palsa or lithalsa systems, with higher DOC concentrations in the highly organic SAS valley ponds, which also had lower pH values due to their elevated humic acid content (Fig 2).

### Methanotroph community composition

Here we used *pmoA* transcripts to determine the composition of the potentially active methanotroph community. This method enabled us to identify the methanotrophs that were most actively participating in methane oxidation in thaw ponds and that were unlikely to be dormant, inactive or dead cells [55]. This community may differ in composition and abundance from the overall methanotrophic community given that there may be a decoupling between DNA and RNA relative representation for some bacterioplankton [56], and in the relative abundance of total versus active taxa in the community.

Our results strongly indicate that the main driver of community composition of potentially active methanotrophs in permafrost thaw waters was landscape type, with marked differences between palsa and lithalsa ponds. This effect was greater than either depth or the extent of permafrost thawing. The overriding influence of landscape is consistent with previous work highlighting its importance for overall bacterial community structure [33,34], and this study shows that there were functional implications for the community differences. The communities of potentially active methanotrophs identified in lithalsa thaw ponds in the KWK valley, which lies in the sporadic permafrost zone, were more similar to the lithalsa ponds found hundreds of km away in the BGR and NAS valleys that lie in colder, less degraded landscapes (discontinuous permafrost) than to the communities in SAS ponds, also in the sporadic permafrost zone, but in an organic-rich peatland palsa landscape. These two classes of thaw pond differ in DOC concentrations, and probably also in DOC composition, and the specific effects of differences in organic carbon substrates on community assembly and functioning will require attention in future studies.

The *pmoA* community profiles in the surface and bottom waters suggest similar potentially active methanotrophs, despite large differences in physico-chemical conditions, including oxygen. This was unexpected relative to results from experimental studies. Microcosm experiments on sediments from Lake Washington showed that different species of methanotrophs predominated under different oxygen tensions [57], with a concommitent shift in the assemblage of microbes linked to the methanotrophs [58]. This might suggest a greater resilience of planktonic communities to variable oxygen regimes in the water column, versus the selection of more specialized genotypes in the sediment environment, where physico-chemical conditions are likely to be more stable.

Much of the total bacterial production in thermokarst lakes and ponds is associated with suspended particles [37], and the interior of such particles could provide anaerobic micro-niches that would allow methanogens to thrive and supply methane to aerobes located closer to the particle surface. We found a significant difference in methanotroph composition between filter size fractions in the SAS palsa valley ponds, but not at the other sites. Particle sizes in the SAS ponds are larger than in other thaw ponds, caused in part by flocculation at the higher DOC concentrations [37], and the size and composition of these particles may influence community assembly processes. Distinct particle-associated microbial communities in coastal marine environments have been found using the same size fractionation protocol as our study [59,60]. In contrast, we found no significant differences between large and small fractions in the lithalsa ponds, which may be due to the same taxa able to move between attached and pelagic lifestyles in lithalsa ponds. However, it cannot be ruled out that clogging of the 3 μm filters would have resulted in retention of some free-living cells in the large fraction.

Type I methanotrophs were the dominant contributors to the community of potentially active methane consuming bacteria in all thaw ponds. This contrasts with reports that boreal and subarctic peatland soils are more typically dominated by Type II taxa [61–64] and yield Type II *pmoA* sequences from DNA and cDNA [62], although other studies have shown a predominance of Type I taxa in some boreal peatlands and fens [65,66]. Type I methanotrophs appear to be more adapted to colder environments, for example in high latitude tundra soils [67,68], and some species such as *Methylobacter tundripalidum* are psychrotolerant [69,70] while other taxa, such as *Methylobacter psychrophilus*, are truly psychrophilic [71]. Studies in soils and sediments have shown that Type II methanotrophs tend to be more abundant and active when temperatures rise to around 20°C [72,73], while Type I methanotrophs are active in colder environments [68,74]. He et al. [75] recorded a shift from Type I to Type II methanotrophs in Arctic lake sediments with increasing temperature, and also a shift in community composition within each type. In temperate lakes, Type I methanotrophs appear to be the dominant contributors to community biomass and activity [76]. They are also more active and in higher abundance in suboxic metalimnetic and hypolimnetic waters [17,76,77], suggesting a preference for low oxygen conditions. Type I dominance in the Quebec subarctic thaw ponds may reflect the prevalence of cold conditions throughout the annual cycle. Even in the SAS ponds, at the warmer southern end of the sampling region, water temperatures remain below 10°C in most of the water column for most of the year, and below 5°C for 7 months each year [53]. Type I methanotrophs might similarly be favored by the low oxygen regime in subarctic thaw ponds, where surface concentrations may be as low as 2.2 mg oxygen L^-1^ (Table 2). The ongoing warming of the subarctic landscape [41] may eventually disrupt these oxygen conditions, leading to changes in the relative abundance of active methanotrophs.

The *pmoA* gene targeted here is the most commonly used functional gene to study aerobic methanotrophs because the enzyme (*particulate methane mono-oxygenase*) is present in almost all methanotroph genera [21] and its phylogeny is congruent with that based on the 16S rRNA gene [76,77]. Soluble *methane mono-oxygenase* (*sMMO*) on the other hand, is restricted to certain species [78] and is reportedly only expressed under copper limitation [79], making it a much less reliable marker to study methanotroph diversity. The primers used here to amplify the *pmoA* transcripts are verified for known alpha- and gamma-proteobacteria methanotrophs but would not have detected *pmoA* related genes from the NC10 phylum [26,80], the genus *Crenothrix* [24], nor for taxa in the Verrucomicrobia [25,81]. We note that previous studies on the bacterial communities in these permafrost thaw ponds based on 16S rRNA genes and 16S rRNA failed to detect members of the NC10 phylum and only one OTU of *Crenothrix*, in low abundance, has been recorded. For the Verrucomicrobia that account for 1 to 6% of the reads of the total bacterial community in the KWK and SAS valleys [34,82], the presence of any methanotrophic taxa in this group would need to be verified with alternative primers.

### Methanotrophic potential activity

Few previous studies have estimated the concentration of *pmoA* transcripts in natural environments, but those published to date imply that the values here could be considered a reasonable proxy of methanotrophic activity. In a boreal wetland, the number of *pmoA* transcripts was positively correlated with CH_4_ oxidation rates [83], and in rice paddy soils, most of the respiration (likely dominated by methane oxidation) occurred in a submillimetre zone that contained a highly active population of methanotrophs, with up to 18 *pmoA* transcripts per cell [84]. Studies based on *pmoA* copy numbers from DNA analysis have also found a positive correlation with methane oxidation rates [66,85], and Tuomivirta et al. [85] note that closer relationships would be predicted with *pmoA* transcripts, as measured here.

Potential methanotrophic activity in the thaw ponds did not follow the pronounced landscape pattern that we observed in community structure. Only ‘year of sampling’ had a significant effect on methanotrophic potential activity, consistent with the large interannual variability in certain limnological properties of these aquatic ecosystems. This means that the functional trait of methanotrophy was expressed independently of species composition, suggesting a certain functional redundancy amongst the different phylotypes of methanotrophs. This is in accord with studies indicating that there can be a high level of functional redundancy in bacterial communities and that gene expression strongly depends on the environment [86,87]. It contrasts, however with a phospholipid fatty acid-stable isotope probe (PLFA-SIP) study, which reported a link between methanotrophic activity and community composition in a forest soil [88].

The PLS analysis indicated that three environmental factors primarily influenced methanotrophic potential activity: total phosphorus concentration, conductivity and total suspended solids. Phosphorus has been shown to enhance microbial CH_4_ oxidation in soil [89], and could increase methanotrophic potential by enabling an overall increase in microbial biomass [90]. The link with TSS suggests that methanotrophic potential activity is related to the presence of particles, which in general are microbial activity hot-spots [91] with oxic/anoxic interfaces [92] that would favour methanotrophs.

The PLS analysis also identified the relative abundance of *Methylocystis* as a factor associated with *pmoA* expression, yet this genus represented only a small fraction of the reads (from non-detectable to a maximum of 5% in the SAS valley). Conversely, the most abundant genus, *Methylobacter*, showed no statistical relationship with putative methanotrophic potential activity. This might reflect how taxa with low proportional abundance in the “rare biosphere” may have a disproportionate effect on key the ecosystem functions [93–95]. Modelling studies suggest that rare species impart ecosystem resilience [96], and that their disappearance could result in decreased ecosystem stability. *Methylocystis* is also known to be acid-tolerant [55,97,98] with a capacity for parallel fermentative metabolism under low oxygen conditions [99]. These features may allow this genus to grow in the more acidic, oxygen-poor conditions of the SAS ponds. The large contribution by *Methylocystis* to potential activity might also be due to an over-representation of *pmoA* transcripts, since some strains have two isozymes of the *pmo* enzyme [100].

Contrary to our original hypothesis, methanotrophic potential activity as estimated by transcript concentrations was not closely linked to oxygen concentration. High concentrations of *pmoA* transcripts were recorded in the hypoxic to anoxic bottom waters in addition to the surface of the ponds, suggesting an unexpected degree of metabolic flexibility. This is consistent with increasing evidence that methanotrophs are micro-aerobic and occur even in anoxic waters. For example, Blees et al. [17] found methanotrophs well below the oxycline of an alpine lake where conditions were supposedly anoxic. They measured methane consumption at this depth, implying that methanotrophs can survive and even grow during periods of prolonged anoxia. Aerobic methanotrophs might be able to sustain methane oxidation in such conditions by coupling activity with phototrophs [101,102] or denitrifiers [103,104]. Physical factors in subarctic thaw ponds could also favor persistence of methanotrophs since methane increases by orders of magnitude down the water column [28], and methanotrophs would therefore be closer to their carbon and energy source towards the bottom of the pond. Additionally, small amounts of oxygen may be intermittently transported to deeper waters when mixing of the epilimnion creates a supply of oxygen close to the thermocline, and internal waves propagate across the oxycline [105] creating intermittent oxygen pulses for the microbes.

The relationship between methanotrophic potential activity and methane concentration depended on depth of sampling: while no trend was observed at the surface, a negative relationship was found between methane concentration and methanotrophic activity in the small fraction for the bottom samples, suggesting depletion of methane by free-living methanotrophs. A negative relationship between CH_4_ fluxes and number of *pmoA* transcripts was observed at a peatland site [106], but in contrast, Kankaala et al. [10] showed a positive linear relationship between methanotrophic activity and CH_4_ in a boreal lake, suggesting that the methanotrophs were limited by CH_4_ concentrations at that site. These contrasting results indicate that the relationship between methanotrophic activity and methane concentration is still poorly understood and may vary among environments.

## Conclusions

The permafrost landscape has a major influence on the type of thaw ponds that develop, and this in turn has a selective effect on the relative contribution of potentially active methanotrophs in the communities, with a marked difference between organic palsa and mineral lithalsa ponds. In both landscape types, the community was dominated by Type I methanotrophs that are characteristic of low-temperature environments. However, methanotrophic potential activity as measured by *pmoA* transcripts did not follow the landscape pattern, and was mainly influenced by total phosphorus, conductivity and total suspended solids, which varied greatly within each pond type. High methanotroph potential activity from *pmoA* transcripts was attributed to the Type II genus *Methylocystis*, which was rare, implying a disproportionate, functional importance of certain taxa. Contrary to expectation, *pmoA* transcripts occurred in hypoxic and anoxic bottom waters as well as in surface oxygenated waters. This implies that methanotrophs are active under a wide range of oxygen conditions and may be resilient to future changes in stratification, mixing and oxygenation.

## Supporting information

S1 File **Fig A in S1 File. Rarefaction curve of the *pmoA***. OTUs were clustered at 93%. **Table A in S1 File. Ponds sampled during the 2012 and 2013 field campaign and the availability of data. Table B in S1 File. Properties of the *pmoA* primers fused with the Trueseq sequencing primers. Table C in S1 File. Physico-chemical properties of the surface and bottom (0.5 m above the sediments) of the sampled ponds**. Temperature (T°C), Conductivity in μS cm^-1^ (Cond), dissolved oxygen in mg L^-1^ (O_2_), pH, total nitrogen in mg L^-1^ (TN), total phosphorus in μg L^-1^ (TP) dissolved organic carbon in mg L^-1^ (DOC), total suspended solids (TSS) in mg L^-1^, Chlorophyll a in μg L^-1^ (Chla), concentration of carbon dioxide in μM (CO_2_) and concentration of methane in μM (CH_4_). -: no data.
(DOCX)
