# Supplementary material for: Diversity and potential activity of methanotrophs in high methane-emitting permafrost thaw ponds
Source: PLoS One. 2017 Nov 28;12(11):e0188223. doi: 10.1371/journal.pone.0188223 (PMC5705078; doi:10.1371/journal.pone.0188223)
Supplement: S1 File — Fig A in S1 File. Rarefaction curve of the pmoA. OTUs were clustered at 93%. Table A in S1 File. Ponds sampled during the 2012 and 2013 field campaign and the availability of data. Table B in S1 File. Properties of the pmoA primers fused with the Trueseq sequencing primers. Table C in S1 File. Physico-chemical properties of the surface and bottom (0.5 m above the sediments) of the sampled ponds. Temperature (T°C), Conductivity in μS cm-1 (Cond), dissolved oxygen in mg L-1 (O2), pH, total nitrogen in mg L-1 (TN), total phosphorus in μg L-1 (TP) dissolved organic carbon in mg L-1 (DOC), total suspended solids (TSS) in mg L-1, Chlorophyll a in μg L-1 (Chla), concentration of carbon dioxide in μM (CO2) and concentration of methane in μM (CH4). -: no data. (DOCX) [file pone.0188223.s001.docx]

S1 Fig A


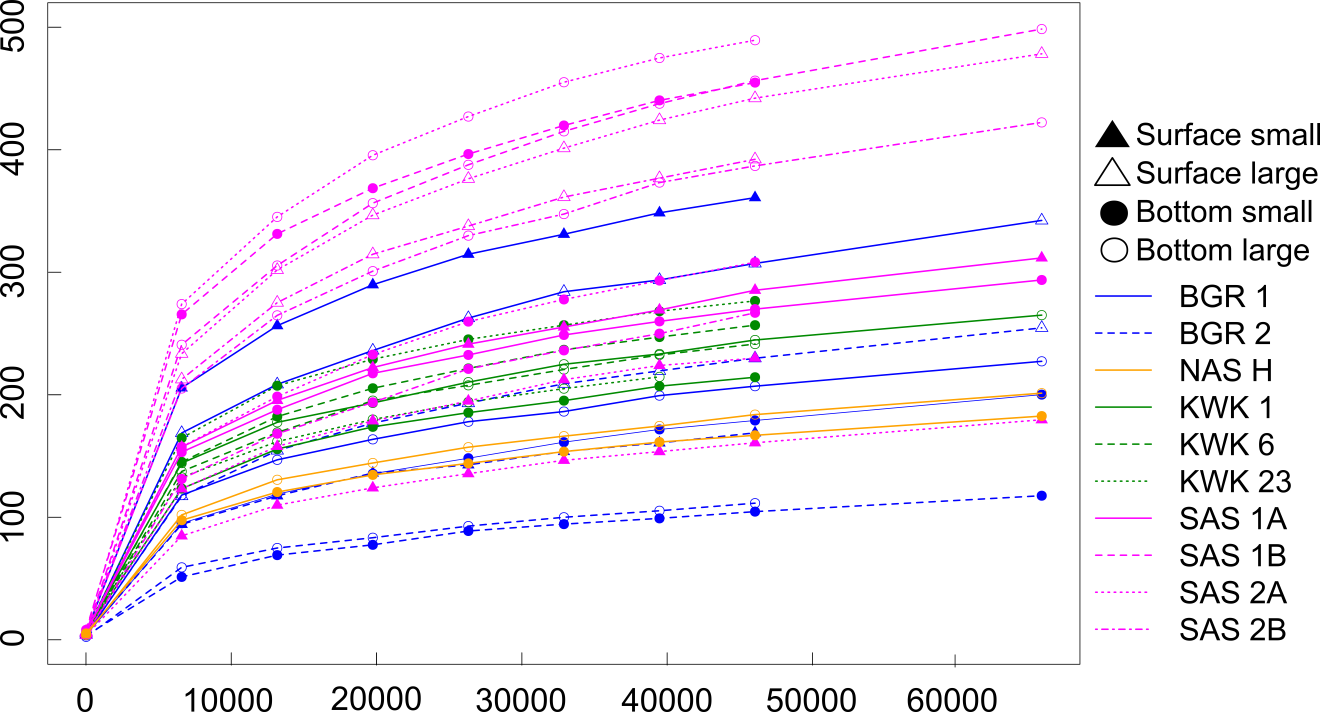


S1 Fig A. Rarefaction curve of the *pmoA*. OTUs were clustered at 93%.

S1 Table A. Ponds sampled during the 2012 and 2013 field campaign and the availability of data.

| Pond | depth | | year | | cDNA | | Illumina sequencing data | | qPCR data | |
| --- | --- | --- | --- | --- | --- | --- | --- | --- | --- | --- |
|  |  |  |  |  | Small | Large |  |  |  |  |
| NAS H | | Surface | | 2012 |  |  |  |  | |  |
|  |  | Bottom | |  | X | X | X | X | |  |
| BGR 1 | | Surface | | 2013 | X | X | X | X | |  |
|  |  | Bottom | |  | X | X | X | X | |  |
| BGR 2 | | Surface | | 2012 | X | X | X | X | |  |
|  |  | Bottom | |  | X | X | X | X | |  |
| KWK 1 | | Surface | | 2012 |  |  |  |  | |  |
|  |  | Bottom | |  | X | X | X | X | |  |
| KWK 6 | | Surface | | 2012 |  |  |  |  | |  |
|  |  | Bottom | |  | X | X | X | X | |  |
| KWK 12 | | Surface | | 2013 |  |  |  |  | |  |
|  |  | Bottom | |  |  |  |  | X | |  |
| KWK 23 | | Surface | | 2012 |  |  |  |  | |  |
|  |  | Bottom | |  | X | X | X | X | |  |
| SAS 1A | | Surface | | 2013 | X |  | X | X | |  |
|  |  | Bottom | |  | X |  | X | X | |  |
| SAS 1B | | Surface | | 2013  2012 |  |  |  |  | |  |
|  |  | Bottom | |  | X | X | X | X | |  |
| SAS 2A | | Surface | | 2013 | X | X |  | X | |  |
|  |  | Bottom | |  | X | X |  | X | |  |
| SAS 2A | | Surface | | 2012 | X | X | X | X | |  |
|  |  | Bottom | |  | X | X | X | X | |  |
| SAS 2B | | Surface | | 2013 | X | X | X | X | |  |
|  |  | Bottom | |  | X | X | X | X | |  |

S1 Table B. Properties of the *pmoA* primers fused with the Trueseq sequencing primers.

| Primer | Targeting region | Trueseq primers | References |
| --- | --- | --- | --- |
| PmoA  169f | *pmoA* | ACACTCTTTCCCTACACGACGCTCTTCCGATCT-GGNGACTGGGACTTCTGG | [77] |
| pmoA 661r | *pmoA* | GTGACTGGAGTTCAGACGTGTGCTCTTCCGATCT- CCGGMGCAACGTCYTTACC | [77] |
| Generic forward second-PCR primer | | AATGATACGGCGACCACCGAGATCTACAC[index1]ACACTCTTTCCCTACACGAC |  |
| Generic reverse second-PCR primer | | CAAGCAGAAGACGGCATACGAGAT[index2]GTGACTGGAGTTCAGACGTGT |  |

S1 Table C. Physico-chemical properties of the surface and bottom (0.5 m above the sediments) of the sampled ponds. Temperature (T°C), Conductivity in µs (Cond), dissolved oxygen in mg L^-1^(O_2_), pH, total nitrogen in mg L^-1^ (TN), total phosphorus in µg L^-1^ (TP) dissolved organic carbon in mg L^-1^ (DOC), total suspended solids (TSS), Chlorophyll *a* in µg L^-1^ (Chla), concentration of carbon dioxide in µM(CO_2_) and concentration en methane in µM (CH_4_). -: no data

| Sample | Depth (m) | T°C | Cond | O_2_ | pH | TN | TP | SRP | DOC | TSS | Chla | CO_2_ | CH_4_ |
| --- | --- | --- | --- | --- | --- | --- | --- | --- | --- | --- | --- | --- | --- |
| NASH-S | 0 | 18.3 | 0.1 | 9.7 | 7.6 | 0.6 | 30.5 | 6.2 | 4.1 | 18.2 | 2.1 | 29.1 | 0.1 |
| NASH-B | 3.4 | 7.1 | 0.1 | 1.7 | 7.3 | 0.6 | 64.8 | 6.3 | 3.7 | 18.3 | 2.0 | - | - |
| BGR1-S | 0 | 20.0 | 0.1 | 9.9 | 8.8 | - | 22.2 | - | - | 1.6 | 0.9 | 22.6 | 1.1 |
| BGR1-B | 3.8 | 9.7 | 0.1 | 3.0 | 7.3 | - | 19.4 | - | - | - | 1.2 | - | - |
| BGR2-S | 0 | 15.0 | 0.2 | 9.4 | 7.3 | 0.5 | 49.1 | 3.4 | 9.3 | 13.1 | 2.4 | 20.2 | 0.4 |
| BGR2-B | 0.8 | 11.0 | 0.4 | 3.5 | 7.2 | 1.2 | 148.9 | 4.5 | 8.7 | 57.4 | 3.8 | 229.2 | 2.6 |
| KWK1-S | 0 | 17.9 | 0.1 | 9.7 | 6.7 | 0.6 | 67.9 | 3.7 | 12 | 20.3 | 10.9 | - | - |
| KWK1-B | 1.8 | 6.4 | 0.2 | 0.5 | 6.2 | 1.1 | 87.8 | 12.6 | 12 | 140.8 | 10.3 | - | - |
| KWK6-S | 0 | 14.0 | 0.1 | 9.9 | 6.4 | 0.4 | 29.8 | 1.3 | 5.2 | 7.3 | 3.3 | - | - |
| KWK6-B | 3 | 8.3 | 0.1 | 1.8 | 6.4 | 0.7 | 99.9 | 1.0 | 5.2 | 16.0 | 27.1 | - | - |
| KWK12-S | 0 | 15.6 | 0 | 8.5 | 7.2 | - | 31.9 | 0.8 | - | 4.6 | 3.7 | 43.7 | 0.2 |
| KWK12-B | 2.2 | 9.3 | 0.1 | 0.3 | 6.4 | - | 49.5 | 5.4 | - | 14.4 | 55 | 2909.3 | 351.3 |
| KWK23-S | 0 | 14.7 | 0.0 | 9.8 | 6.4 | 0.4 | 57.1 | 5.5 | 7.8 | 6.4 | 1.9 | - | - |
| KWK23-B | 3.2 | 4.4 | 0.3 | 0.4 | 6.1 | 2.7 | 170.5 | 133.6 | 10.9 | 73.6 | 7.2 | - | - |
| SAS1A-S | 0 | 18.5 | - | - | - | - | 28.1 | 3.3 | 10.3 | 2.8 | 12.6 | - | - |
| SAS1A-B | 2 | 10.7 | - | - | - | - | - | 4.6 | 9.6 | 11.2 | - | - | - |
| SAS1B-S | 0 | 17.3 | 0.1 | 5.4 | 6.5 | - | 36.1 | - | 14.8 | 13.4 | 3.8 | - | - |
| SAS1B-B | 0.8 | 9.5 | 0.1 | 1.7 | 5.9 | 1.8 | 29.1 | 3.0 | 16.2 | 33.1 | 3 | - | - |
| SAS2A-S | 0 | 19.9 | 0.1 | 5.8 | 6.2 | 0.7 | 10.9 | 3.0 | 14.9 | 2.6 | 1.4 | 470.8 | 3.4 |
| SAS2A-B | 2.4 | 4.6 | 0.3 | 0.3 | 5.6 | 1.6 | 41.5 | 4.1 | 18.9 | - | 18.1 | 1954.9 | 101.7 |
| SAS2A-Sb | 0 | 18.5 | 0.0 | 2.2 | 6.5 | - | 26.2 | 2.5 | 14.8 | 1 | 2 | 382.9 | 1.9 |
| SAS2A-Bb | 2.6 | 4.5 | 0.3 | 0.2 | 6.5 | - | 29.6 | - | 15.4 | 11.2 | 14.9 | 5229.8 | 322.9 |
| SAS2B-S | 0 | 16.4 | 0.1 | 5.2 | 6.8 | - | 24.1 | - | 15.2 | - | 8.6 | 290.0 | 3 |
| SAS2B-B | 2 | 6.1 | 0.1 | 0.2 | 6.4 | - | 58.2 | 3 | 16.1 | 1.7 | 2.7 | 3872.2 | 292 |
